# Mitochondrially Targeted Gene Therapy Rescues Visual Loss in a Mouse Model of Leber’s Hereditary Optic Neuropathy

**DOI:** 10.3390/ijms242317068

**Published:** 2023-12-02

**Authors:** Tsung-Han Chou, Zixuan Hao, Diego Alba, Angelina Lazo, Gabriele Gallo Afflitto, Jeremy D. Eastwood, Vittorio Porciatti, John Guy, Hong Yu

**Affiliations:** Bascom Palmer Eye Institute, University of Miami Miller School of Medicine, Miami, FL 33136, USA; tchou@med.miami.edu (T.-H.C.); zxh424@miami.edu (Z.H.); dea83@miami.edu (D.A.); axl1444@miami.edu (A.L.); ggallo@miami.edu (G.G.A.); jde91@med.miami.edu (J.D.E.); jguy@med.miami.edu (J.G.)

**Keywords:** LHON, MTSAAV, human *ND4*, gene therapy

## Abstract

Leber’s hereditary optic neuropathy (LHON) is a common mitochondrial genetic disease, causing irreversible blindness in young individuals. Current treatments are inadequate, and there is no definitive cure. This study evaluates the effectiveness of delivering wildtype human NADH ubiquinone oxidoreductase subunit 4 *(hND4*) gene using mito-targeted AAV(MTSAAV) to rescue LHOH mice. We observed a declining pattern in electroretinograms amplitudes as mice aged across all groups (*p* < 0.001), with significant differences among groups (*p* = 0.023; Control vs. LHON, *p* = 0.008; Control vs. Rescue, *p* = 0.228). Inner retinal thickness and intraocular pressure did not change significantly with age or groups. Compared to LHON mice, those rescued with wildtype *hND4* exhibited improved retinal visual acuity (0.29 ± 0.1 cy/deg vs. 0.15 ± 0.1 cy/deg) and increased functional hyperemia response (effect of flicker, *p* < 0.001, effect of Group, *p* = 0.004; Interaction Flicker × Group, *p* < 0.001). Postmortem analysis shows a marked reduction in retinal ganglion cell density in the LHON group compared to the other groups (Effect of Group, *p* < 0.001, Control vs. LHON, *p* < 0.001, Control vs. Rescue, *p* = 0.106). These results suggest that MTSAAV-delivered wildtype *hND4* gene rescues, at least in part, visual impairment in an LHON mouse model and has the therapeutic potential to treat this disease.

## 1. Introduction

Mitochondrial dysfunction underlies a large number of diseases and aging processes [1,2,3,4,5]. Many mitochondria-related disorders are the result of point mutations or deletions in the mitochondrial genome [6,7,8,9]. Among these, Leber’s Hereditary Optic Neuropathy (LHON) stands out as the most common primary mitochondrial genetic disease. It manifests as sudden, severe, and irreversible vison loss, predominantly affecting children and young adults. More than 90% of LHON cases are caused by one of three point mutations in the mitochondrial DNA (mtDNA): m.11778G>A, m.3460G>A, and m.14484T>C. These mutations, respectively, affect subunits ND4, ND1, and ND6, which are crucial components of the respiration complex I [10,11,12,13,14].

Pharmacological therapies for LHON, much like in other mitochondrial diseases, have proven to be insufficient, and there is no FDA-approved therapy available currently. Gene therapy approaches have been explored as potential treatment options for LHON [15,16,17,18]. Among these approaches, allotopic expression has advanced to human testing, specifically for m.11778G>A mutation. Clinical trials for this approach are being conducted in China (NCT01267422) [19,20,21,22], France (NCT02064569, NCT02652767, NCT02652780, NCT034061104) [23,24,25,26,27], and the USA (NCT02161380) [28], with the French trial currently in phase 3 trials being conducted in the USA and Europe. While these clinical trials have shown some degree of visual improvement in LHON patients, the overall efficacy has been limited, as per the natural history of the disease. Also, unexpected similar improvements in the untreated eye have complicated the assessment [23,25,28]. As a result, new and innovative approaches are needed to enhance treatment outcomes for LHON patients and provide more significant visual recovery.

Viruses have the ability to traverse the mitochondrial double membrane to access the inner matrix and deliver DNA inside the organelle [29]. Yu et al. have demonstrated that fusing a mitochondrial targeting sequence (MTS) to the capsid of adeno-associated virus (MTS–AAV) can redirect the virus to mitochondria rather than the nucleus [30]. Using the MTS–AAV, we successfully delivered the wildtype human *ND4* gene to LHON cybrids homoplasmic for the m.11778G>A mutation and rescued the cells from defective respiration. We also explored its potential in the adult rodent visual system, where it effectively prevented visual loss and optic nerve atrophy induced by the mutant [R340H]ND4.

Our subsequent studies further demonstrated the versatility of this approach. Introducing the mutant *hND4* gene into mouse zygotes, we generated bona fide LHON mitomice carrying human *ND4*/m.11778G>A mutation. The translated hND4 protein assembled into host respiratory complex I, leading to decreased respiratory chain function and increased oxidative stress [31]. This mitomice model recapitulated the human form of LHON with progressive loss of retinal ganglion cell (RGC) function, as assessed by pattern electroretinogram (PERG) as well as RGC and optic nerve degeneration. RGC functional and structural losses were largely reversed with an intraocular injection of MTS–AAV expressing wildtype human *ND4*. In a more recent study, we used the same approach to deliver human *ND1*/m.3460G>A [32] and *ND6*/m.14484T>C [33] into mouse eyes. The delivered gene induced functional and structural RGC loss. Importantly, our findings revealed that the mitochondrially delivered DNA, compared to allotopic ND4 delivery, showed superior efficacy in rescuing functional and structural RGC loss in LHON mice over a span of 15 months [34].

In the present study, we comprehensively assessed, in LHON mice induced by intravitreal injection of mutant *hND4*, the rescue effects of intravitreally injected MTS–AAV-delivered wild-type *hND4*. Assessment included PERG amplitude/latency, PERG visual acuity, PERG adaptation to flicker-induced metabolic stress, RGC/axon density and integrity, as well as mitochondrial integrity. These studies are part of a preclinical testing of MTS–AAV-delivered *ND4*, and the results obtained from this research will help to gain regulatory approval for this therapy for future human testing.

## 2. Results

### 2.1. Wildtype hND4 Rescues RGC Dysfunction Induced by Mutant hND4

**Figure 1:**
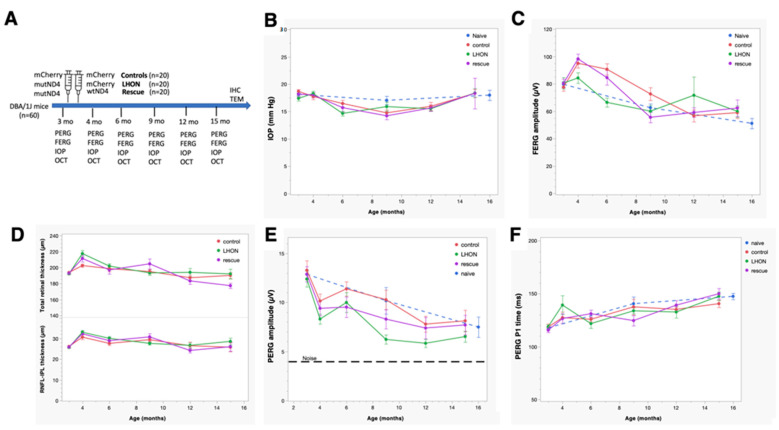
Mito-targeted *wtND4* rescue *mutND4*-induced RGC dysfunction. (**A**) Study design and timeline for the retinal structure and function follow-up. (**B**) Pre-injection and post-injection IOPs were similar in the study groups. (**C**) FERG amplitudes temporarily increased in all study groups after intravitreal injections and then returned to physiological level. (**D**) Inner retina thickness and total retinal thickness were temporarily increased in all study groups after intravitreal injections and then returned to baseline levels. (**E**) PERG amplitudes temporarily decreased in all study groups and progressively recovered the physiological level in Control and Rescue study groups, but not in the LHON group. (**F**) PERG latencies physiologically increased with age in all study groups.

RGCs are the most vulnerable cell type in patients with LHON mutations; we, therefore, wanted to assess whether MTS–AAV-delivered wildtype *hND4* gene could effectively counteract RGC dysfunction induced by mutant *hND4* allele. We conducted the study using 60 DBA/1J mice aged 3 months, which were randomly divided into 3 groups, with 20 mice per group. As illustrated in Figure 1A, two of the groups were injected with MTS–AAV/*hND4G11778A-mCherry* into the vitreous, inducing LHON phenotype. The third group served as a control (Control) and received MTS–AAV/*mCherry* injections. Two days later, one of the LHON groups was treated with MTS–AAV/*hND4-mCherry* to rescue the phenotype (Rescue), while the other LHON group received MTS–AAV/*mCherry* as a disease control (LHON). For the control group (Control), we performed a second injection of MTS–AAV/*mCherry*. Additionally, we included a group of mice that were not injected with any substances as a naïve control (Naïve, n = 4).

As the first step, we wanted to detect whether double injections had any discernible impact on intraocular pressure (IOP). We observed significant age-related changes in IOP in all four groups of mice (Factor age, *p* < 0.01). However, these changes were relatively small, ranging from approximately 3 to 5 mmHg. There were no significant differences between the groups (Factor group, *p* = 0.76; Age × Group, *p* = 0.26). Importantly, the IOP changes observed over time were within the range of normal physiological variations seen in naïve mice, suggesting that the double injections did not cause abnormal or extreme fluctuations in IOP levels (Figure 1B).

Considering that the visual pathway begins with the retinal photoreceptors and the signal transmits to the retinal ganglion cells, we then measured outer retinal function, using flash electroretinogram (FERG). As shown in Figure 1C, the amplitude of FERG exhibited a noteworthy reduction as age increased (Factor age, *p* < 0.001). However, this decrease did not display any notable distinctions between the groups (Factor Group, *p* = 0.55) despite the time course not completely overlapping (Age × Group, *p* = 0.003). Intriguingly, although the decline in FERG amplitude within the study groups was akin to that observed in naïve mice, there was a temporary increase in all study groups at the first post-injection measurement.

Next, to detect if the injection induced any change in retinal structure, we used spectral-domain optical coherence tomography (SD-OCT) to quantify the thickness of the inner retinal layers, encompassing the nerve fiber layer (RNFL), ganglion cell layer (RGC), and inner plexiform layer (IPL) (Figure 1D upper panel), as well as the overall retinal thickness (Figure 1D lower panel). The GEE analysis showed that both the total and inner retinal thicknesses displayed age-related changes that were comparable to that in the FERG. Specifically, a significant age-related decline was evident (Factor Age, *p* < 0.001); however, there was no significant difference between Groups (Factor Group, *p* > 0.3) and the time course was not overlapping (Age × Group, *p* < 0.005). Additionally, a transient thickening of the retina was observed across all study groups at the first post-injection measurement, coinciding with an increase in FERG amplitude. This phenomenon could be potentially caused by sterile inflammation, as reported in post-operative cataract patients [35,36].

Then, we determined whether MTS–AAV-delivered wildtype *hND4* could reverse RGC dysfunction induced by mutant *hND4* allele in mice using PERGs, a sensitive electrophysiologic measure for RGC function. Before the intravitreal injection, conducted when the mice were at 3 months of age, no discernible differences were observed in PERG amplitude between the naïve and the injected mice of each group. However, the GEE analysis unveiled a progressive decline in PERG amplitude across all groups, albeit with differing patterns between the groups (effect of Age, *p* < 0.001; effect of Group, *p* = 0.025; Age × Group, *p* = 0.74).

Post hoc comparison revealed that the PERG amplitude in the Control group was larger than that in the LHON group (*p* = 0.009), while no substantial difference was noted compared to the Rescue group (*p* = 0.12). It is important to note that the progressive decline in PERG amplitude within the Control group almost coincided with a similar decline observed in naïve mice. This indicates that the age-related decline in PERG amplitude in the control mice was primarily related to physiological decline occurring in DBA/1J mice and was not attributed to the influence of double intravitreal injection of *mCherry*. However, the double intravitreal injections did lead to a temporary drop in PERG amplitude across all groups at the first post-injection measurement, which subsequently displayed partial recovery in later measurements. Due to the inherent physiological decline, the PERG amplitude of all groups tended to converge at 15 months of age, as the dynamic range of response (the difference between the amplitude in naïve mice and noise) decreased with increasing age. In dynamic range units, the group difference at the 15-month time point was substantial: the LHON group exhibited an approximate 40% reduction in normal PERG amplitude, whereas the control and rescue groups maintained a normal amplitude (Figure 1E).

Furthermore, GEE showed that the PERG latency increased with ages in all study groups (Factor Age, *p* < 0.001, Factor Group, *p* = 0.57, Age × Group, *p* = 0.58). Notably, the age-related PERG latency increase in the study groups paralleled a similar increase in the naïve group (Figure 1F).

Collectively, these findings provide compelling evidence that intravitreal injection of the mutant *hND4* only induced RGC dysfunction, characteristic of LHON disease in humans. Moreover, this induced phenotype appears to be subject to long-term rescue through a subsequent intravitreal injection of the wildtype allele.

### 2.2. Wildtype hND4 Rescues Loss of Visual Acuity Induced by Mutant hND4

**Figure 2:**
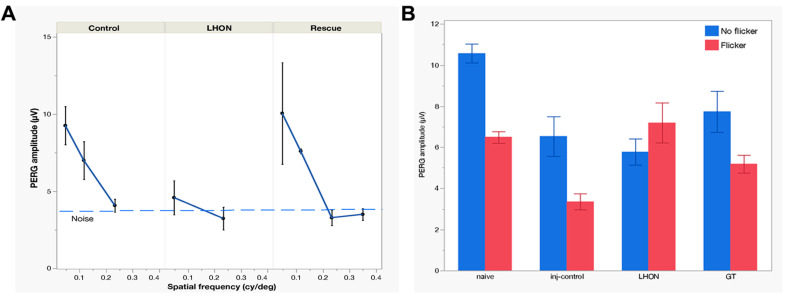
Wildtype *ND4* improves the visual acuity. (**A**) The PERG signal decreases with increasing spatial frequency. The linear extrapolation of PERG amplitude to the noise level represents the retinal acuity, which is higher in the Control group (0.242 cy/deg) and in the Rescue group (0.293 cy/deg), compared to the LHON group (0.153 cy/deg). (**B**) Flickering light superimposed on the PERG stimulus causes reduction of PERG amplitude (autoregulatory adaptation), which is present in both Naïve and Control group (34% amplitude change). Flicker adaptation is lost in the LHON group, but it is restored (30%) in the Rescue group.

Visual acuity loss is the most obvious manifestation of LHON. It can be objectively measured in mice using optomotor (OMR) reflex [37,38]. However, less-pigmented mice, such as DBA/1J, exhibit a deficiency in their head-tracking response and reflex during optokinetic tests, likely due to a developmental abnormality in ocular melanin synthesis. This condition leads to retinal underdevelopment and misrouting of the visual pathway [39]. In this study, we opted for a surrogate measure of visual acuity by using the PERG, a method detailed in prior publications [40]. PERG responses were recorded at multiple spatial frequencies. As spatial frequency increased, the PERG amplitude exhibited a gradual decline, eventually reaching the threshold of background noise level (signal-to-noise ratio = 1). The intersection of PERG amplitude with the noise level represented the spatial resolution of the retinal output (PERG acuity). As shown in Figure 2A, in a subgroup of mice 15 months old, the PERG-derived visual acuity was 0.242 cy/deg in the Control group (n = 8), 0.153 cy/deg in the LHON group (n = 6), and 0.293 cy/deg in the Rescue group (n = 6).

Altogether, this experiment unveils a notable visual acuity decrement of approximately 0.2 LogMAR (equivalent to roughly two lines on the Snellen Chart) in LHON mice compared to controls. Importantly, this acuity loss was effectively prevented by the application of wildtype hND4 in the rescued group.

### 2.3. Wildtype hND4 Rescues Loss of RGC Metabolic Autoregulation Induced by Mutant hND4

It is well-known that flicking light induces rapid dilation of retinal vessels, a phenomenon known as functional hyperemia. In both mice and humans, this flicker-induced functional hyperemia is associated with an autoregulatory response of RGCs, consisting of a slow reduction of PERG amplitude to a plateau—an occurrence termed PERG adaptation [41,42,43]. PERG adaptation is found to be compromised in human [41] and mouse glaucoma [43], indicating a deficiency in the ability of RGCs to autoregulate in response to a metabolically challenging stimulus. In a subgroup of Naïve (n = 10), Control (n = 7), LHON (n = 8), and Rescue (n = 8) mice, we have measured flicker-induced PERG adaptation by sequentially recording PERG responses with superimposed flicker at two distinct frequencies: 101 Hz (not detectable) and 11 Hz (visible). As adaptive responses are typically variable, we combined data collected in the post-injection age range of 5–15 months in order to increase the sample size. Figure 2B shows that the transition from non-flicker to flicker produced a marked reduction in PERG amplitude, totaling 33% in Naïve mice and 34% in Control mice, indicating normal autoregulation [41]. In contrast, the LHON group exhibited a loss of flicker adaptation, pointing to a malfunction in the autoregulatory mechanism. Remarkably, in the Rescue group, the restoration of flicker adaptation was achieved to a magnitude of 30%, and this rescue effect was statistically significant (effect of flicker, *p* < 0.001, effect of Group, *p* = 0.004; Interaction Flicker × Group, *p* < 0.001).

### 2.4. Wildtype hND4 Rescues Loss of RGCs Induced by Mutant hND4

**Figure 3:**
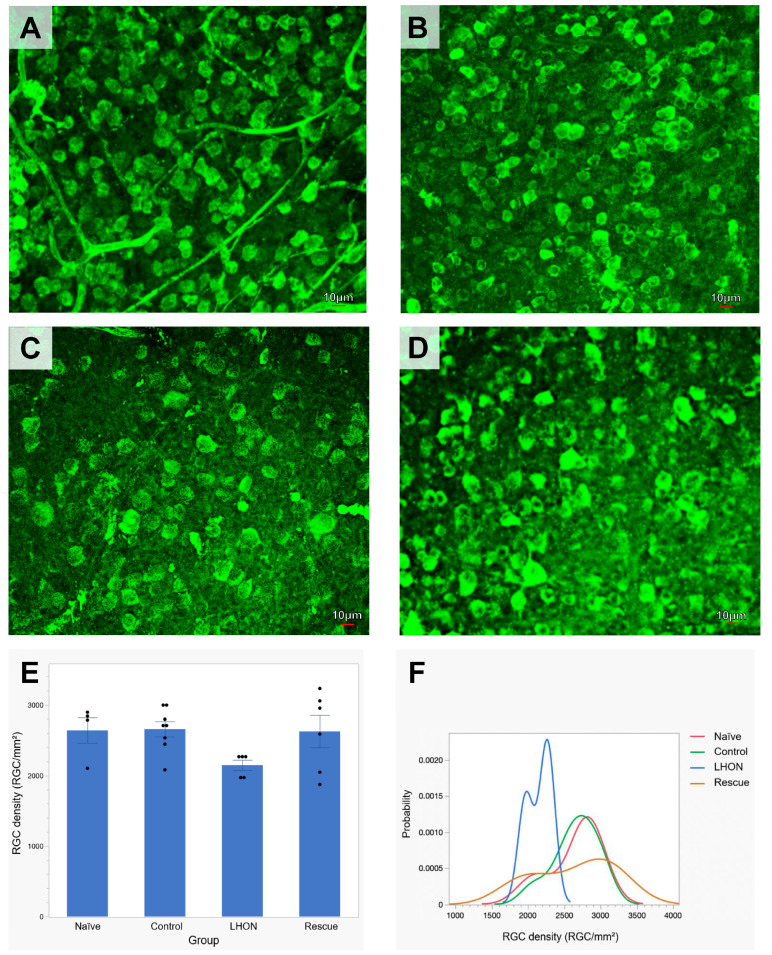
Wildtype *ND4* prevents RGC loss in LHON mice. (**A**–**D**) Representative immunostaining of retinal whole-mount using RBPMS (a pan-RGC marker) showed RGCs distribution in Naïve (**A**), Control (**B**), LHOH (**C**), and Rescue (**D**) mice. (**E**) The mean RGC density at the endpoint is lower in the LHON group compared to Naïve, Control, and Rescue groups. (**F**) The probability distribution of RGC density in the LHON group is shifted to the left compared to overlapping distributions of Control and Naive groups. The Rescue group appears to have a bimodal distribution, with one peak in the LHON range and another peak in the range of Controls. Scale bar = 10 μm.

To further assess the rescue effects of delivered wildtype *hND4,* we performed post-mortem histological analysis at 15 months post-intravitreal injection. The average density of RBPMS-positive RGCs was quantified in flat-mounted retinae in groups of the Naïve (n = 3, Figure 3A), Control (n = 5, Figure 3B), LHON (n = 4, Figure 3C), and Rescue (n = 4, Figure 3D). Multiple measures from the same mice were accounted for in the GEE analysis. The effect between the groups was highly significant (*p* < 0.001). Specifically, a significant decline in RGC density was evident in the LHON group compared to the Control group (*p* < 0.001), while RGC density in the Rescue group was similar to that in either Control or Naïve mice (*p* = 0.106, Figure 3E).

To provide visual representations of RGC density distributions across the different groups, we expressed RGC densities collected in all retinal samples as probability density functions. As shown in Figure 3F, RGC distribution of Control mice exhibited an overlap with that of Naïve mice, while the RGC distribution of LHON mice displayed a narrower overlap than that of Control mice and was markedly shifted towards smaller densities. The RGC distribution of Rescue mice appears to be bimodal, with one subpopulation of RGC densities in the Control range and another subpopulation in the LHON range. Altogether, these results suggest that the delivered wildtype *hND4* can rescue, at least in part, the loss of RGCs induced by mutant *hND4*.

### 2.5. Wildtype hND4 Rescues Optic Atrophy Induced by Mutant hND4

**Figure 4:**
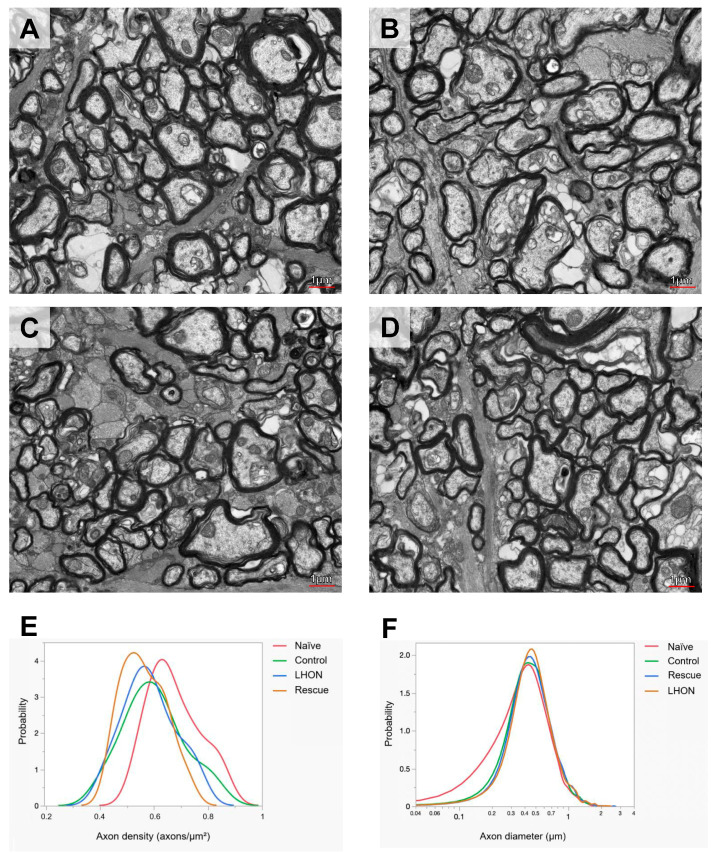
Wildtype *ND4* protects optic nerve degeneration. (**A**–**D**) Representative transmission electron micrographs of the retrobulbar optic nerve showed the axon distribution in Naïve (**A**), Control (**B**), LHON (**C**), and Rescue (**D**) mice. (**E**) The distribution of axon density shows higher density in the Naïve group compared with the other three groups. (**F**) The main distribution of axon diameter is similar in all groups, but the Naïve group has a tail of axons smaller than 0.3 µm. Scale bar = 1 μm.

To evaluate the rescue efficacy of optic atrophy, we performed ultrastructural analysis using transmission electron microscopy (TEM) 15 months after intravitreal injection. Three optic nerves were analyzed from each group of the Naïve (Figure 4A), Control (Figure 4B), LHON (Figure 4C), and Rescue (Figure 4D). Axon density was calculated from axon counts on eight sections of each optic nerve. Since the sample was limited, results were descriptive and expressed as probability density functions of all measurements for each group. We found that distribution of axon density in all study groups was shifted toward smaller axon density, compared to that in age-matched Naïve mice. However, there was no obvious difference in axon density distributions between Control, LHON, and Rescue groups (Figure 4E).

Next, axon size was measured on about 2000 axons of each optic nerve. The distribution of axon size in all study groups had lost a subpopulation of axons smaller than 0.3 µm, compared to that in age-matched Naïve mice. Otherwise, the distribution of axon sizes in Control, LHON, and Rescue groups appeared to be very similar (Figure 4F).

**Figure 5:**
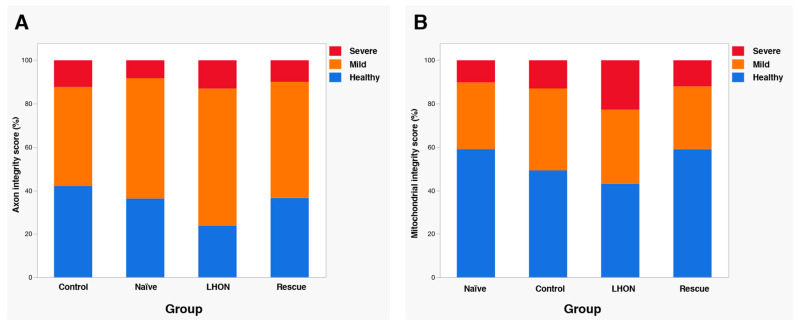
Wildtype *ND4* preserves mitochondrial and RGC axonal integrity. Integrity scoring for the axon (**A**) and mitochondria in the optic nerve (**B**). LHON rescue group has similar healthy axons and mitochondria as the age Control and negative Control groups. (N = 5 per group, 15-month-old).

To further evaluate the morphological of axons and mitochondria, we used a well-established scoring system previously reported [44,45,46]. This scoring system allowed us to assign a “health score” to each sample, categorizing it as follows: 1 (no or minimal changes), 2 (definite but moderate changes), and 3 (severe changes). The scoring was performed by two independent observers who were blinded to the study groups of the optic nerves. Analysis of both axon (Figure 5A) and mitochondria scores (Figure 5B) clearly demonstrate a higher frequency of moderate-to-severe ultrastructural changes within the LHON group in comparison to the Naïve and Control groups. However, in the Rescue group, the distribution of health score approached that observed in Naïve and Control mice. Altogether, these results indicate that the wildtype *hND4* rescues, at least in part, the ultrastuctural axonal and mitochondrial changes induced by the mutant *hND4*.

## 3. Discussion

Current treatments for LHON remain insufficient, leaving the disease without a definitive cure. Several therapeutic strategies are in development to manage LHON, including genetic therapies, antioxidant and neurotrophic therapies, promotion of mitochondrial biogenesis, mitochondrial replacement therapy, and stem cell-based approaches. Among these, allotopic gene therapy has advanced to human clinical trials for the m.11778G>A mutation in China (NCT01267422) [19], France (NCT02064569) [23], and the USA (NCT02161380) [28]. Despite the observation of visual improvement among participants in these clinical trials, the majority of individuals still experience low vision, leading them to be categorized as legally blind, especially those with visual loss for more than one year [23,28,47]. The limited therapeutic efficacy observed might be due to the hydrophobic nature of the ND4 protein, making its import and sorting across the mitochondrial membrane challenging when produced remotely, such as in the cytosol [48,49,50]. MTS–AAV overcomes these challenges by directly delivering the gene of interest (*ND4*) into the mitochondria. Our previous reports have successfully demonstrated the expression of the human *ND4* gene delivered by MTS–AAV in mouse RGCs and its integration into the mitochondrial respiratory complex I [31]. Building upon this foundation, our current study further assessed the rescue efficacy of MTS–AAV-delivered wildtype *hND4* in an LHON mouse model. Our comprehensive evaluation included visual acuity, RGC metabolic autoregulation, RGC function, as well as the integrity of axons and mitochondria in the optic nerve.

PERG spatial resolution has proven to be a valuable tool for assessing visual acuity in wildtype and mutant mice [30]. PERG acuity measurements reflect the spatial resolution of the retinal output [49,50] and align with those obtained through other methods, such as optomotor response (OMR) and pattern visual evoked potential (PVEP) [51]. In this study, we used PERG acuity as mice of the DBA strain, such as albino mice, do not have an OMR reflex. Our results showed that the PERG acuity of LHON mice was lower than the acuity of control mice. However, the PERG acuity of mice rescued with wildtype *hND4* was similar to the PERG acuity of control mice.

Flickering light increases metabolic demand in the inner retina and induces functional hyperemia [34,35,51]. When flickering light is superimposed on the PERG stimulus, the PERG signal progressively declines to a plateau lower than the initial amplitude by about 30% [41]. PERG adaptation to flicker represents a physiological autoregulatory mechanism that is lost in dysfunctional RGCs [41]. Our results show that flicker-induced PERG adaptation is lost in LHON mice but is regained in rescued mice. These results suggest that wildtype *hND4* preserved an autoregulatory mechanism of RGCs that depends on retinal metabolism.

The LHON mutation has been identified as a trigger for a defect in the assembly or stability of complex I within LHON cybrids. The degree of this complex I deficiency seems to correlate with the specific mtDNA backgrounds, such as the U5a haplotype rather than the more common J haplotype, associated with LHON mutations, rather than being tied to heteroplasmic load [52]. Notably, around 15% of LHON patients exhibit heteroplasmy for primary LHON mutations [53,54,55,56]. Strikingly, these patients present an identical disease phenotype to those who are homoplasmic for the mutations.

In our study using transgenic mitomice, the introduction of mutant *hND4* amounted to 20% of the mouse *ND4* levels; nevertheless, these mice displayed LHON hallmarks [31]. This result strongly suggests a dominant negative effect originating from the mutant *hND4* in the mice. This observation potentially explains the partial rescue of the phenotype induced by mutant *hND4* in this study. Further investigations will be needed to confirm the findings using a primate LHON model, considering their high anatomic resemblance and the presence of ND4 homologous to humans.

In conclusion, we assessed the rescue efficacy of MTS–AAV-delivered wildtype *hND4* in an LHON mouse model. The delivered gene demonstrated its potential to counteract the harmful effects of mutant *hND4* in mice. Specifically, it displayed the ability to prevent RGC loss, halt RGC dysfunction, restore RGC metabolic autoregulation, uphold visual acuity, and preserve the integrity of axons and mitochondria.

Results from this study will serve as additional validation for our IND-enabling dataset, specifically aimed at rescuing rodent models of LHON through this mitochondrially targeted approach. Our ongoing efforts will persist in assessing the toxicology and biodistribution of MTS–AAV-mediated *hND4* delivery, with the explicit goal of formulating an efficacious treatment strategy for future clinical trials addressing LHON cases carrying the *ND4G11778A* mutation.

## 4. Materials and Methods

### 4.1. Plasmids and AAVs

sc-HSPCSB-hND4-mCherry was constructed as previously described [30,31]. In brief, human *ND4* gene was fused in frame with *FLAG*, and mitochondrial-encoded *Cherry* (*mCherry*) was cloned into scAAV backbones under the control of the mitochondrial heavy strand promoter (*HSP*), including three upstream conserved sequence blocks (*HSPCSB*), where *ND4FLAG* is followed by *mCherry* with a stop codon between two genes. *mCherry* cloned in the same scAAV backbone was used as a control (sc-HSPCSB-mCherry). Mutant human *ND4G11778A* gene was cloned into the same AAV backbone under the control of *HSP* (sc-HSP-hND4G11778A-mCherry) and used for making LHON mouse model. The resultant plasmids were purified using Qiagen endotoxin-free maxiprep (Qiagen, Hilden Germany) and then packaged with the VP2COX8 plus VP1, VP3, and helper plasmid PXX6 into recombinant virus: MTS–AAV/*hND4-mCherry*, MTS–AAV/*mCherry*, and MTS–AAV/*hND4G11778A-mCherry*.

### 4.2. Animals and Intravitreal Injections (IVI)

All animal procedures were performed abiding by the National Institutes of Health Guide for Care and Use of Laboratory Animals and the ARVO Statement for the use of Animals in Ophthalmic and Vision Research. Three-month-old DBA/1J (D1) mice (n = 60) were sedated by the IP injection of ketamine (100 mg/mL, 1.5 mL), xylazine (20 mg/mL, 1.5 mL), and injectable water (7 mL) mixture for the pattern electroretinograms (PERG), flash electroretinograms (FERG), and spectral-domain optical coherence tomography (SD-OCT) baseline recordings. After the PERG baseline recordings, we randomized the mice into three groups (n = 20 per group) to make the averaged PERG similar between the groups. Then, we delivered intravitreally the MTS–AAV/*hND4G11778A-mCherry* (6E9 vg/eye) into both eyes in 40 D1 mice and divided them randomly into LHON (n = 20) and Rescue (n = 20) groups. The LHON group (n = 20) received MTS–AAV/*mCherry* (6E9 vg/eye), and the Rescue group (n = 20) received MTS–AAV/*hND4-mCherry* (6E9 vg/eye). A Control group (n = 20) received two IVI of MTS–AAV/*mCherry* (6E9 vg/eye).

### 4.3. IOP, PERG, FERG, and SD-OCT

Intraocular pressure (IOP) was assessed using a rebound tonometer (iCare TONOLAB tonometer, Vantaa, Finland) within the initial 5 min of the anesthesia period.

RGC function was assessed by Pattern Electroretinogram (PERG), using a commercially available instrument (Jorvec Corp. Miami, FL, USA) to simultaneously record responses from both eyes, as previously described in detail [41]. In brief, ketamine-/xylazine-anesthetized mice were gently restrained in a holder, allowing unobstructed vision, and kept at a constant body temperature of 37.0 °C using a feedback-controlled heating pad controlled by a rectal probe. Pupils were undilated and small (<1 mm), which insured a large depth of focus. PERG signals were recorded from a subcutaneous stainless-steel needle (Grass, West Warwick, RI, USA) placed in the snout. The reference and ground electrodes were similar needles placed medially on the back of the head and at the root of the tail, respectively. Visual stimuli were presented at each eye independently from a 10 cm distance and consisted of contrast-reversal of gratings (0.05 cycles/deg, 98% contrast) generated on two light-emitting diode (LED) tablets (15 × 15 cm square field, 800 cd/m^2^ mean luminance), alternating at slightly different frequencies around 1 Hz (OD, 1.016 Hz; OS, 1.008 Hz). Independent PERG signals from each eye were retrieved using one-channel continuous acquisition and phase-locking average over 372 epochs for each eye. PERG amplitude was measured peak-to-trough using software that automatically detected the positive peak and the negative trough in the PERG waveform (typically the P1 peak to the N2 trough). Noise responses were obtained by computing the difference between even and odd epochs.

To have a corresponding index of outer retinal function, a light-adapted flash electroretinogram (FERG) was also recorded with undilated pupils in response to strobe flashes of 20 cd/m^2^/s superimposed on a steady background light of 12 cd/m^2^ and presented within a Ganzfeld bowl. Under these conditions, rod activity is largely suppressed, while cone activity is minimally suppressed.

Retinal images were visualized with Heidelberg spectral-domain optical coherence tomography (SD-OCT). After the OCT images were taken, we used the IOWA Reference Algorithms OCT Explore software (Version 3.8.0) to quantitatively analyze the mouse retina layer of thickness [57,58].

### 4.4. PERG-Based Visual Acuity

Retinal visual acuity in mice was measured when they were 12-month-old by semi-automatic PERG recordings with multiple trials in different spatial frequencies. The peak-to-trough (P1 to N2) PERG amplitudes at different spatial frequencies were automatically measured together with the background noise. The PERG amplitude typically decreases with increasing spatial frequency. The PERG acuity was calculated by extrapolating PERG amplitude to the noise level [40].

### 4.5. Flicker-Induced PERG Adaptation

**Figure 6:**
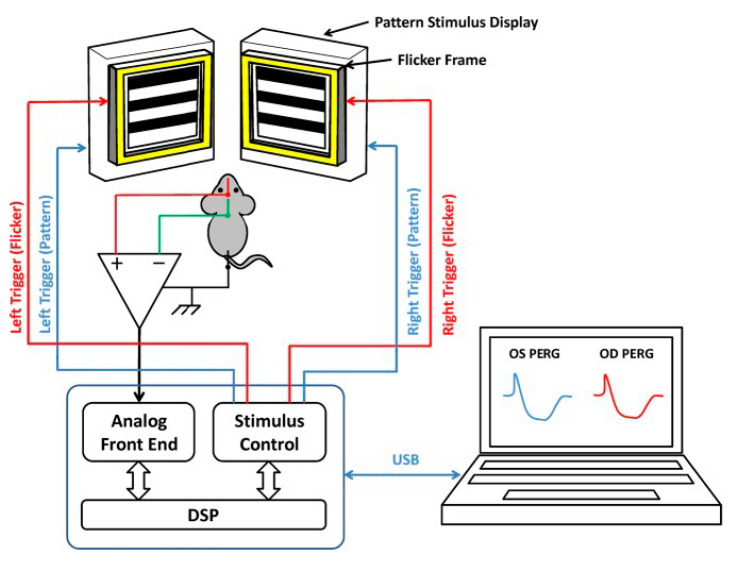
Block diagram for binocular PERG recording. Pattern stimuli with two slightly different reversal rates are generated on two identical LED displays and presented separately to each eye. PERG signals are recorded continuously by means of subcutaneous needle electrodes (active, snout; reference, back of the head; ground, tail) and fed to one-channel acquisition system. The PERG signals for each eye are desynchronized by phase locking averaging method and PERG waveforms are displayed on the screen of a laptop that controls the stimulation/acquisition box.

To assess the effect of flicker on PERG, a flickering field was superimposed on the patterned stimulus as previously described [41,42,43] (Figure 6). The patterned LED display was surrounded by an LED square frame (internal size 15 × 15 cm, external size 18 × 18 cm) to generate flickering light. The flickering frame had the same mean luminance as the patterned field and could be modulated (square-wave, 50% duty cycle) at either 101 Hz or 11 Hz at constant mean luminance. These frequencies were asynchronous with the pattern-reversal frequencies and did not contribute to the PERG waveform. At 101 Hz, flickering light could not be perceived by human observers. PERG + 101 Hz flicker (baseline) and PERG +11 Hz flicker (test) were recorded in sequence.

### 4.6. Immunostaining

At 15 months old, mouse retinas were harvested for RGC counting (RBPMS immunostaining) and mouse optic nerves were harvested for axon counting and axon mitochondria scoring (Transmission Electron Microscopy, TEM, a JEOL JEM-1400, Tokyo, Japan). Scoring axon and mitochondria were measured by double-blinded observers using a three-score system (healthy, mild, severe status).

### 4.7. Statistical Analysis

Data analysis was performed using Generalized Estimating Equation (GEE, IBM SPSS statistics Ver. 26), an unbiased non-parametric method to analyze longitudinal correlated data that also accounts for inclusion of multiple measurements from the same subject [59,60].

In the longitudinal analyses, dependent variables were PERG, FERG, IOP, and OCT, and predictor variables were age (3, 6, 9, 12, 15 months) and treatment group (Control, LHON, Rescue). Main effects (Age, Group) and interaction between Age and Group were computed, as well as pairwise combinations between Age and Group. An additional longitudinal group of naïve mice (3, 9, 16 months of age, n = 4) was included for comparison but not incorporated in the GEE main analysis.

## Data Availability

The original contributions presented in the study are included in the article. Further inquiries can be directed to the corresponding authors.
